# Supplementary material for: Conditional chemoconnectomics (cCCTomics) as a strategy for efficient and conditional targeting of chemical transmission
Source: eLife. 2024 Apr 30;12:RP91927. doi: 10.7554/eLife.91927 (PMC11060718; doi:10.7554/eLife.91927)
Supplement: Figure 3—source data 3. [file elife-91927-fig3-data3.zip › Figure 3 source data3 part 2.zip!/Figure 3-Source data 3-part2/1053-1-CRISPResso_Report_mpeqg9da.zip!/CRISPResso_on_1053_4/1a.Read_barplot.pdf]

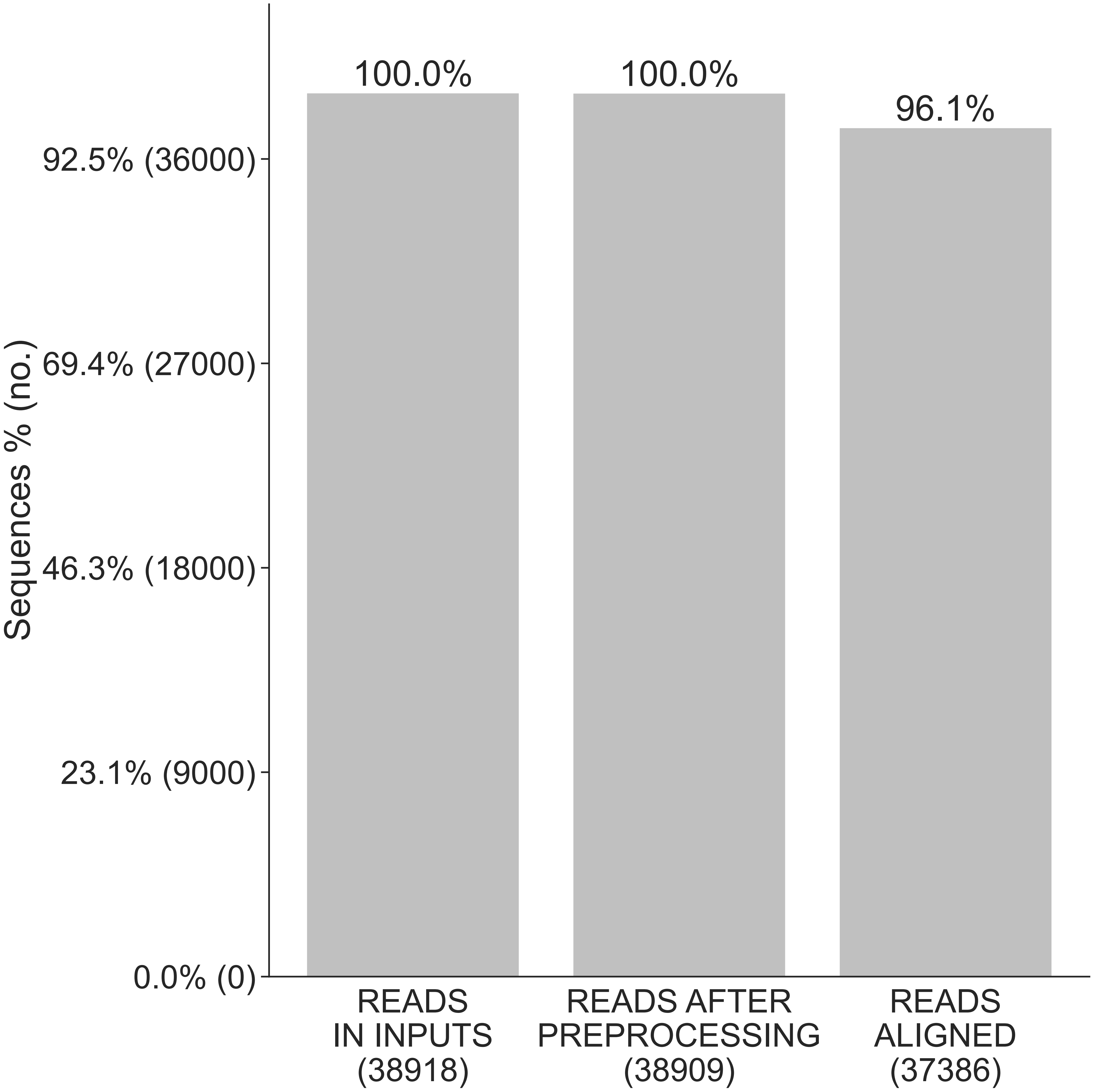
Sequences % (no.)
92.5% (36000)
69.4% (27000)
46.3% (18000)
23.1% (9000)
0.0% (0)
100.0%
100.0%
96.1%
READS IN INPUTS (38918)
READS AFTER PREPROCESSING (38909)
READS ALIGNED (37386)
